# Severe Hypoglycemia due to Isolated ACTH Deficiency in Children: A New Case Report and Review of the Literature

**DOI:** 10.1155/2011/784867

**Published:** 2011-03-30

**Authors:** Michael Y. Torchinsky, Robert Wineman, George W. Moll

**Affiliations:** Department of Pediatrics (M.Y.T., G.W.M.), and Department of Radiology (R.W.), University of Mississippi Medical Center, Jackson, MS 39216, USA

## Abstract

Isolated ACTH deficiency causes life-threatening severe hypoglycemia. A 7-year-old girl with hypoglycemia due to this rare disorder is described. Our patient had undetectable plasma ACTH repeatedly and cortisol 0 mcg/dl before and after ACTH 1-24 stimulation. There was no evidence of other pituitary hormone deficiency. Glucocorticoid replacement therapy resulted in resolution of all symptoms and normalization of blood glucose. Previously published data on isolated ACTH deficiency in children is summarized. Review of the literature showed that the prevalence of this condition could be underestimated in the neonatal period and in Prader-Willi syndrome. Isolated ACTH deficiency occurs in older children as well as in neonates.

## 1. Introduction

Isolated ACTH deficiency is a life-threatening disorder which causes severe hypoglycemia. Described in a pediatric patient by Hung and Migeon in 1968 [1], it can present during the first year of life as result of a specific genetic mutation, such as a pro-opiomelanocortin (POMC) or T-box transcription factor (TPIT) mutation [2–5], or later in childhood due to unknown factors [1, 6–10]. We report a new case of this unusual condition and summarize data in children published in the literature. 

## 2. Case Report

A 7-year-old girl was admitted to the hospital due to episode of unconsciousness following a fall from a scooter. Her blood glucose was 19 mg/dL and urine ketones were positive. She regained consciousness after treatment with intravenous glucose. It turned out that six months earlier she had seizures and blood glucose 21 mg/dl during a febrile illness treated in intensive care unit. Analysis of her cerebrospinal fluid ruled out meningitis, and electroencephalogram was normal. Fatigue, decreased appetite, and increased irritability were noted by parents since age 4 years. 

**Figure 1:**
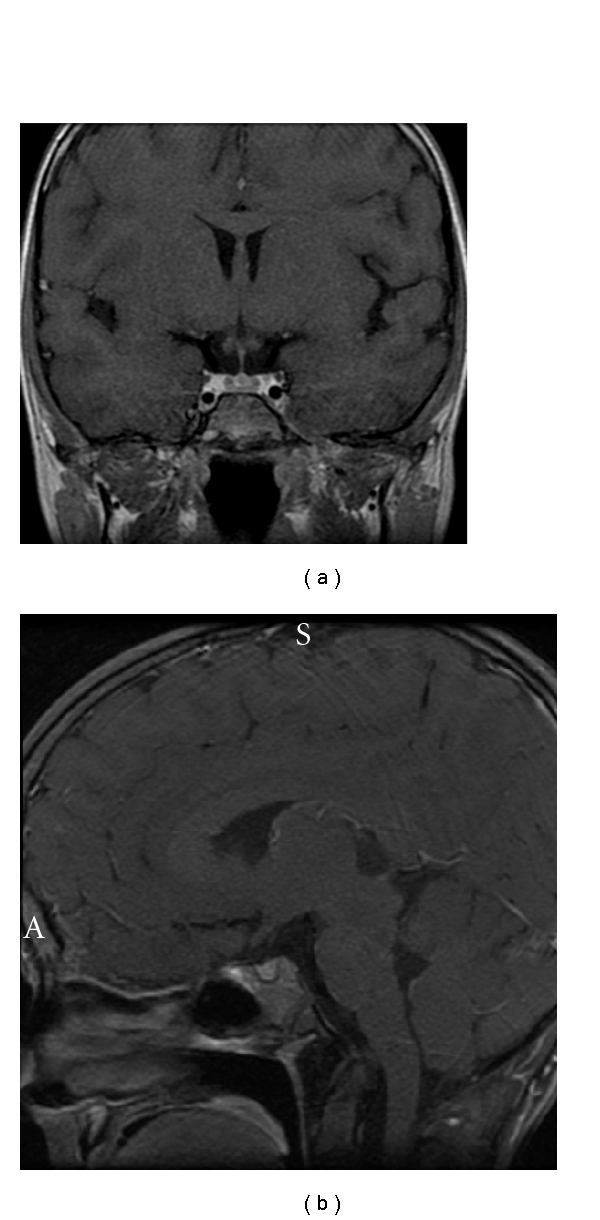
Magnetic resonance imaging scan of the brain. A coronal section shows a tiny cyst in the midline of the pituitary gland (a). A sagittal section demonstrates that the tiny cyst is at the posterior margin of the anterior pituitary (b).

Her physical exam was unremarkable; there was no hyperpigmentation. Height was 123.1 cm (50 < *P* < 75%), weight 22.6 kg (25 < *P* < 50%), and BMI 14.9 kg/m^2^ (25 < *P* < 50%). BP was normal. Laboratory evaluation showed that serum TSH was 2.20 mU/L, free T4 1.67 ng/dL, T3 1.42 ng/mL, prolactin 10.2 ng/mL, ACTH <5 pg/mL, cortisol 0.0 ug/dL, DHEAS <15 ug/dL, IGF-1 296 ng/mL, plasma renin activity 2.60 ng/mL/hr, and normal serum electrolytes. Repeat morning ACTH was <5 pg/mL, and cortisol 0.0 ug/dL. ACTH 1–24 stimulation test showed cortisol 0.0 ug/dL at 45 min after Cortrosyn 0.250 mg injection. CRH stimulation test demonstrated plasma ACTH <5 pg/mL at 15 min, 30 min, 45 min, and 60 min after Acthrel 1 mcg/kg intravenous injection. Head MRI scan without and with contrast using pituitary protocol demonstrated a tiny 2-3 mm cyst localized in the midline at the posterior margin of the anterior pituitary (Figure 1). 

Detailed evaluation of adrenal function and pituitary function demonstrated that the child had isolated ACTH deficiency. Genetic cause for this disorder in our patient remained unknown. Treatment with Hydrocortisone 16 mg/m^2^/day divided in three doses given by mouth resulted in resolution of all symptoms and normalization of blood glucose. The patient remained symptoms free and had normal IGF-1, TSH, and free T4 on clinic followup one year after the diagnosis. 

## 3. Discussion

Our patient had undetectable cortisol levels before and after Cortrosyn stimulation, which indicated that her hypoglycemia was due to glucocorticoid deficiency. Very low ACTH despite undetectable cortisol showed that glucocorticoid deficiency was secondary to ACTH deficiency. Lack of hyperpigmentation and absence of electrolyte abnormalities, such as hyperkalemia and hyponatremia, normal blood pressure, and plasma renin activity provided evidence that ACTH-independent mineralocorticoid function was preserved, which further supported diagnosis of secondary adrenal insufficiency. 

Growth hormone secretion was sufficient to maintain normal height and IGF-1, and other pituitary hormones were normal, in particular, TSH and prolactin, which was consistent with isolated ACTH deficiency. Absent ACTH response to CRH stimulation indicated that defect in ACTH production was localized in the pituitary and was not due to hypothalamic dysfunction. 

To our knowledge, this is the first time that isolated ACTH deficiency was associated with a tiny cyst in the midline of the pituitary. In contrast to somatotroph, lactotroph, and thyrotroph cells scattered throughout the anterior pituitary, corticotroph cells are mainly located in its central region [11]. The tiny cyst close to that region might be due to an unknown genetic factor that caused isolated ACTH deficiency or could be an incidental finding [12, 13]. 

In comparison with isolated deficiencies of other pituitary hormones, such as idiopathic GH deficiency, gonadotropin deficiency, and central hypothyroidism, isolated ACTH deficiency is uncommon, except for ACTH suppression after treatment with exogenous glucocorticoids [14]. ACTH deficiency usually occurs in combination with other pituitary hormone deficiencies as part of hypopituitarism [11]. A possibility of lymphocytic hypophysitis should be considered particularly if there is pituitary enlargement, a mass, or thickening of the pituitary stalk [15, 16]. 

Isolated ACTH deficiency during the first year of life was reported in thirty-three children. Five of them had POMC mutations, eighteen had TPIT mutations, and ten patients had unknown genetic defect [2–5]. Among twenty-two families of children with isolated ACTH deficiency, where detailed history was obtained, six families (>25% of cases) suffered an unexpected neonatal death of the patient's sibling [4, 5]. POMC and TPIT mutations were inherited as autosomal recessive traits [2, 4]. 

Loss-of-function mutations of the POMC gene resulted in a complete loss of POMC-derived ACTH, a-MSH and b-endorphin, and caused secondary hypocortisolism, red hair, pale skin and early-onset severe obesity. Among five children with this syndrome, four presented with hypoglycemic seizures during the first weeks of life, and the fifth patient at age 12 months [2, 3]. 

Inactivating TPIT mutations disrupted terminal differentiation of corticotroph cells specialized in POMC gene expression and presented with severe hypoglycemia, seizures, and failure to thrive in neonatal period in all cases, although the diagnosis was occasionally delayed until age 2 years. Among eighteen patients with isolated ACTH deficiency due to a TPIT mutation, eleven had prolonged cholestatic jaundice [4, 5]. 

High prevalence of central adrenal insufficiency was recently discovered in Prader-Willi syndrome. Fifteen out of twenty-five patients with this syndrome had subnormal ACTH response on Metyrapone test, indicative of isolated ACTH deficiency [9]. These striking findings unveiled a potential reason for very high annual death rate in children with Prader-Willi syndrome (3%), who are at risk for unexpected fatal outcomes during mild-to-moderate respiratory tract infections, fatal apnea, and bathtub drowning [9]. Treatment with Hydrocortisone during stress was recommended unless central adrenal insufficiency was ruled out by Metyrapone test [9, 17]. Further study of hypothalamic-pituitary-adrenal axis in Prader-Willi syndrome is needed [18].

Diagnosis of isolated ACTH deficiency in otherwise healthy children after infancy is very rare. There were only six such cases between ages 2 and 9 years published in the literature [1, 6–8, 10]. All children presented with severe hypoglycemia. In four out of six cases, hypoglycemic seizures or loss of consciousness occurred during stress of febrile illness [1, 7, 8, 10]. The cause of ACTH deficiency was unknown. There were singular reports of isolated ACTH deficiency in association with Kabuki syndrome and multiple congenital malformations [19, 20]. 

## 4. Conclusions

We conclude that isolated ACTH deficiency is an unusual diagnosis and its prevalence could be underestimated, in particular in the neonatal period and in children with Prader-Willi syndrome. Clinicians should be aware that this rare condition occurs in older children as well as in neonates.
